# Global estimates on the number of people blind or visually impaired by diabetic retinopathy: a meta-analysis from 2000 to 2020

**DOI:** 10.1038/s41433-024-03101-5

**Published:** 2024-06-27

**Authors:** Vision Loss Expert Group of the Global Burden of Disease Study, Katie Curran, Tunde Peto, Jost B. Jonas, David Friedman, Judy E. Kim, Janet Leasher, Ian Tapply, Arthur G. Fernandes, Maria Vittoria Cicinelli, Alessandro Arrigo, Nicolas Leveziel, Serge Resnikoff, Hugh R. Taylor, Tabassom Sedighi, Seth Flaxman, Mukkharram M. Bikbov, Tasanee Braithwaite, Alain Bron, Ching-Yu Cheng, Monte A. Del Monte, Joshua R. Ehrlich, João M. Furtado, Gus Gazzard, M. Elizabeth Hartnett, Rim Kahloun, John H. Kempen, Moncef Khairallah, Rohit C. Khanna, Van Charles Lansingh, Kovin S. Naidoo, Vinay Nangia, Michal Nowak, Konrad Pesudovs, Pradeep Ramulu, Fotis Topouzis, Mitiadis Tsilimbaris, Ya Xing Wang, Ningli Wang, Rupert R. A. Bourne, the GBD 2019 Blindness and Vision Impairment Collaborators, Katie Curran, Tunde Peto, Rupert Bourne, Janet L. Leasher, Jost B. Jonas, David S. Friedman, Judy E. Kim, Arthur G. Fernandes, Bright Opoku Ahinkorah, Hamid Ahmadieh, Ayman Ahmed, Ahmad Samir Alfaar, Louay Almidani, Hubert Amu, Sofia Androudi, Jalal Arabloo, Aleksandr Y. Aravkin, Mulu Tiruneh Asemu, Ahmed Y. Azzam, Nayereh Baghcheghi, Freddie Bailey, Mehmet Firat Baran, Mainak Bardhan, Till Winfried Bärnighausen, Amadou Barrow, Pankaj Bhardwaj, Mukharram Bikbov, Tasanee Braithwaite, Paul Svitil Briant, Katrin Burkart, Luis Alberto Cámera, Kaleb Coberly, Omid Dadras, Xiaochen Dai, Amin Dehghan, Berecha Hundessa Demessa, Mengistie Diress, Thanh Chi Do, Thao Huynh Phuong Do, Klara Georgieva Dokova, Bruce B. Duncan, Michael Ekholuenetale, Muhammed Elhadi, Mohammad Hassan Emamian, Mehdi Emamverdi, Hossein Farrokhpour, Ali Fatehizadeh, Lorenzo Ferro Desideri, João M. Furtado, Mesfin Gebrehiwot, Fariba Ghassemi, Mesay Dechasa Gudeta, Sapna Gupta, Veer Bala Gupta, Vivek Kumar Gupta, Billy Randall Hammond, Mehdi Harorani, Hamidreza Hasani, Golnaz Heidari, Mehdi Hosseinzadeh, John J. Huang, Sheikh Mohammed Shariful Islam, Nilofer Javadi, Aida Jimenez-Corona, Mohammad Jokar, Charity Ehimwenma Joshua, Vidya Kadashetti, Himal Kandel, Hengameh Kasraei, Rimple Jeet Kaur, Sudarshan Khanal, Zahra Khorrami, Hamid Reza Koohestani, Kewal Krishan, Stephen S. Lim, Mohammed Magdy Abd El Razek, Vahid Mansouri, Andrea Maugeri, Tomislav Mestrovic, Awoke Misganaw, Ali H. Mokdad, Hamed Momeni-Moghaddam, Sara Momtazmanesh, Christopher J. L. Murray, Hadush Negash, Uchechukwu Levi Osuagwu, Shahina Pardhan, Jay Patel, Shrikant Pawar, Ionela-Roxana Petcu, Hoang Tran Pham, Mohsen Pourazizi, Ibrahim Qattea, Mosiur Rahman, Umar Saeed, Amirhossein Sahebkar, Mohammad Amin Salehi, Maryam Shayan, Aminu Shittu, Jaimie D. Steinmetz, Yao Tan, Fotis Topouzis, Aristidis Tsatsakis, Muhammad Umair, Theo Vos, Hong Xiao, Yuyi You, Mikhail Sergeevich Zastrozhin, Zhi-Jiang Zhang, Peng Zheng

**Affiliations:** 1 Centre for Public Health, Queens University Belfast, Belfast, Northern Ireland; 2 Department of Ophthalmology, Medical Faculty Mannheim, Heidelberg University, Heidelberg, Germany; 3 Mass Eye and Ear, Harvard Medical School, Boston, MA USA; 4 University of Texas Southwestern Medical Center, Dallas, TX USA; 5 Nova Southeastern University College for Optometry, Fort Lauderdale, FL USA; 6 Department of Ophthalmology, Cambridge University Hospitals, Cambridge, UK; 7 Federal University of Sao Paolo, Sao Paolo/SP, Brazil; 8 University of Calgary, Calgary/AB, Canada; 9 School of Medicine, Vita-Salute San Raffaele University, Milan, Italy; 10 Department of Ophthalmology, IRCCS San Raffaele Scientific Institute, Milan, Italy; 11 Scientific Institute San Raffaele Hospital, Vita-Salute University, Milan, Italy; 12 University of Poitiers, Poitiers, France; 13 CHU de Poitiers, Poitiers, France; 14 Brien Holden Vision Institute, Sydney, NSW Australia; 15 School of Optometry and Vision Sciences, Faculty of Medicine, University of New South Wales, Sydney, NSW Australia; 16 School of Population and Global Health, University of Melbourne, Carlton, VIC Australia; 17 Vision and Eye Research Institute, Anglia Ruskin University, Cambridge, UK; 18 Department of Computer Science, University of Oxford, Oxford, UK; 19 Ufa Eye Research Institute, Ufa, Russia; 20 School of Life Course and Population Sciences, King’s College London, London, UK; 21 The Medical Eye Unit, Guy’s and St Thomas’ NHS Foundation Trust, London, UK; 22 University Hospital, Dijon, France; 23 National University of Singapore, Singapore, Singapore; 24 Singapore Eye Research Institute, Singapore, Singapore; 25 University of Michigan, Ann Arbor, MI USA; 26 Kellogg Eye Center, Ann Arbor, MI USA; 27 Institute for Social Research, University of Michigan, Ann Arbor, MI USA; 28 Department of Ophthalmology and Visual Sciences, University of Michigan, Ann Arbor, MI USA; 29 Ribeirão Preto Medical School, University of São Paulo, São Paulo, Brazil; 30 Institute of Ophthalmology UCL & NIHR Biomedical Research Centre, London, UK; 31 Stanford University, Stanford, CA USA; 32 Associated Ophthalmologists of Monastir, Monastir, Tunisia; 33 Department of Ophthalmology, Harvard University, Boston, MA USA; 34 Eye Unit, MyungSung Medical College, Addis Ababa, Ethiopia; 35 Department of Ophthalmology, Addis Ababa University, Addis Ababa, Ethiopia; 36 Sight for Souls, Bellevue, WA USA; 37 Fattouma Bourguiba University Hospital, University of Monastir, Monastir, Tunisia; 38 Allen Foster Community Eye Health Research Centre, Gullapalli Pratibha Rao International Centre for Advancement of Rural Eye care, L.V. Prasad Eye Institute, Hyderabad, India; 39 Brien Holden Eye Research Centre, L.V. Prasad Eye Institute, Banjara Hills, Hyderabad, India; 40 School of Optometry and Vision Science, University of New South Wales, Sydney, NSW Australia; 41 University of Rochester, School of Medicine and Dentistry, Rochester, NY USA; 42 HelpMeSee, Instituto Mexicano de Oftalmologia, Santiago de Querétaro, Mexico; 43 University of Miami, Miami, FL USA; 44 University of Utah, Salt Lake City, UT USA; 45 African Vision Research Institute, University of KwaZulu-Natal (UKZN), Durban, South Africa; 46 Suraj Eye Institute, Nagpur, India; 47 Institute of Optics and Optometry, University of Social Science, 121 Gdanska str., Lodz, 90-519 Poland; 48 Medicine & Health, University of New South Wales, Sydney, NSW Australia; 49 John Hopkins Wilmer Eye Institute, Baltimore, MD USA; 50 1st Department of Ophthamology, Medical School, Aristotle University of Thessaloniki, Ahepa Hospital, Thessaloniki, Greece; 51 University of Crete Medical School, Giofirakia, Greece; 52 Beijing Institute of Ophthamology, Beijing Tongren Hospital, Capital Medical University, Beijing Ophthamology and Visual Sciences Key Laboratory, Beijing, China; 53 Beijing Institute of Ophthamology, Beijing Tongren Eye Center, Beijing Tongren Hospital, Capital Medical University, Beijing, China; 54 Centre for Public Health, Queen’s University Belfast, Belfast, UK; 55 Vision and Eye Research Unit, Anglia Ruskin University, Cambridge, UK; 56 College of Optometry, Nova Southeastern University, Fort Lauderdale, FL USA; 57 Institute of Molecular and Clinical Ophthalmology Basel, Basel, Switzerland; 58 Department of Ophthalmology, Heidelberg University, Mannheim, Germany; 59 Mass Eye and Ear Department of Ophthalmology, Harvard Medical School, Boston, MA USA; 60 Department of Ophthalmology and Visual Sciences, Medical College of Wisconsin, Milwaukee, WI USA; 61 Department of Ophthalmology and Visual Sciences, Federal University of São Paulo, São Paulo, Brazil; 62 School of Public Health, University of Technology Sydney, Sydney, NSW Australia; 63 Ophthalmic Research Center, Shahid Beheshti University of Medical Sciences, Tehran, Iran; 64 Department of Ophthalmology, Shahid Beheshti University of Medical Sciences, Tehran, Iran; 65 Institute of Endemic Diseases, University of Khartoum, Khartoum, Sudan; 66 Swiss Tropical and Public Health Institute, University of Basel, Basel, Switzerland; 67 Department of Ophthalmology, University of Leipzig Medical Center, Leipzig, Germany; 68 Department of Ophthalmology, Charité Medical University Berlin, Berlin, Germany; 69 Wilmer Eye Institute, Johns Hopkins University School of Medicine, Baltimore, MD USA; 70 Doheny Image Reading and Research Lab (DIRRL) - Doheny Eye Institute, University of California Los Angeles, Los Angeles, CA USA; 71 Department of Population and Behavioural Sciences, University of Health and Allied Sciences, Ho, Ghana; 72 Department of Medicine, University of Thessaly, Volos, Greece; 73 Health Management and Economics Research Center, Iran University of Medical Sciences, Tehran, Iran; 74 Department of Applied Mathematics, University of Washington, Seattle, WA USA; 75 Institute for Health Metrics and Evaluation, University of Washington, Seattle, WA USA; 76 Department of Health Metrics Sciences, School of Medicine, University of Washington, Seattle, WA USA; 77 Department of Public Health, Debre Tabor University, Debre Tabor, Ethiopia; 78 Department of Neurovascular Research, Nested Knowledge, Inc., Saint Paul, MN USA; 79 Faculty of Medicine, October 6 University, 6th of October City, Egypt; 80 Department of Nursing, Saveh University of Medical Sciences, Saveh, Iran; 81 Big Data Institute - GRAM Project, University of Oxford, Oxford, UK; 82 Vocational School of Technical Sciences, Batman University, Batman, Türkiye; 83 Miami Cancer Institute, Baptist Health South Florida, Miami, FL USA; 84 Heidelberg Institute of Global Health (HIGH), Heidelberg University, Heidelberg, Germany; 85 T.H. Chan School of Public Health, Harvard University, Boston, MA USA; 86 Department of Epidemiology, University of Florida, Gainesville, FL USA; 87 Department of Public & Environmental Health, University of The Gambia, Brikama, The Gambia; 88 Department of Community Medicine and Family Medicine, All India Institute of Medical Sciences, Jodhpur, India; 89 School of Public Health, All India Institute of Medical Sciences, Jodhpur, India; 90 Epidemiology Department, Ufa Eye Research Institute, Ufa, Russia; 91 Ophthalmology Department, Moorfields Eye Hospital NHS Foundation Trust, London, UK; 92 International Centre for Eye Health, London School of Hygiene & Tropical Medicine, London, UK; 93 Internal Medicine Department, Hospital Italiano de Buenos Aires (Italian Hospital of Buenos Aires), Buenos Aires, Argentina; 94 Board of Directors, Argentine Society of Medicine, Buenos Aires, Argentina; 95 Department of Addiction Medicine, Haukland University Hospital, Bergen, Norway; 96 Department of Global Public Health and Primary Care, University of Bergen, Bergen, Norway; 97 School of Medicine, Isfahan University of Medical Sciences, Isfahan, Iran; 98 USAID-JSI, Jimma University, Addis Ababa, Ethiopia; 99 Department of Human Physiology, University of Gondar, Gondar, Ethiopia; 100 Department of Medicine, Pham Ngoc Thach University of Medicine, Ho Chi Minh City, Viet Nam; 101 Department of Medicine, Can Tho University of Medicine and Pharmacy, Can Tho, Viet Nam; 102 Department of Social Medicine and Health Care Organisation, Medical University “Prof. Dr. Paraskev Stoyanov”, Varna, Bulgaria; 103 Postgraduate Program in Epidemiology, Federal University of Rio Grande do Sul, Porto Alegre, Brazil; 104 Department of Epidemiology and Medical Statistics, University of Ibadan, Ibadan, Nigeria; 105 Faculty of Public Health, University of Ibadan, Ibadan, Nigeria; 106 Faculty of Medicine, University of Tripoli, Tripoli, Libya; 107 Ophthalmic Epidemiology Research Center, Shahroud University of Medical Sciences, Shahroud, Iran; 108 Department of Ophthalmology, University of California Los Angeles, Los Angeles, CA USA; 109 School of Medicine, Tehran University of Medical Sciences, Tehran, Iran; 110 Endocrinology and Metabolism Research Institute, Non-Communicable Diseases Research Center (NCDRC), Tehran, Iran; 111 Department of Environmental Health Engineering, Isfahan University of Medical Sciences, Isfahan, Iran; 112 University Eye Clinic, University of Genoa, Genoa, Italy; 113 Division of Ophthalmology, University of São Paulo, Ribeirão Preto, Brazil; 114 Department of Environmental Health, Wollo University, Dessie, Ethiopia; 115 Ophthalmology Department, Tehran University of Medical Sciences, Tehran, Iran; 116 Department of Clinical Pharmacy, Haramaya University, Harar, Ethiopia; 117 Toxicology Department, Shriram Institute for Industrial Research, Delhi, India; 118 School of Medicine, Deakin University, Geelong, VIC Australia; 119 Faculty of Medicine Health and Human Sciences, Macquarie University, Sydney, NSW Australia; 120 Brain and Behavioral Sciences Program, University of Georgia, Athens, GA USA; 121 Department of Nursing, Arak University of Medical Sciences, Arak, Iran; 122 Department of Ophthalmology, Iran University of Medical Sciences, Karaj, Iran; 123 Independent Consultant, Santa Clara, CA USA; 124 Institute of Research and Development, Duy Tan University, Da Nang, Viet Nam; 125 Department of Computer Science, University of Human Development, Sulaymaniyah, Iraq; 126 Department of Ophthalmology and Visual Science, Yale University, New Haven, CT USA; 127 Institute for Physical Activity and Nutrition, Deakin University, Burwood, VIC Australia; 128 Sydney Medical School, University of Sydney, Sydney, NSW Australia; 129 Student Research Committee, School of Medicine, Isfahan University of Medical Sciences, Isfahan, Iran; 130 Department of Pediatrics, Isfahan University of Medical Sciences, Isfahan, Iran; 131 Department of Ocular Epidemiology and Visual Health, Institute of Ophthalmology Foundation Conde de Valencia, Mexico City, Mexico; 132 Directorate General of Epidemiology, Mexico City, Mexico; 133 Zoonoses Research Center, Islamic Azad University, Karaj, Iran; 134 Department of Clinical Sciences, Jahrom University of Medical Sciences, Jahrom, Iran; 135 Department of Economics, National Open University, Benin City, Nigeria; 136 Department of Oral and Maxillofacial Pathology, Krishna Vishwa Vidyapeeth (Deemed to be University), Karad, India; 137 Save Sight Institute, University of Sydney, Sydney, NSW Australia; 138 Sydney Eye Hospital, South Eastern Sydney Local Health District, Sydney, NSW Australia; 139 Eye Research Center, Iran University of Medical Sciences, Tehran, Iran; 140 Health Policy Research Center, Shiraz University of Medical Sciences, Shiraz, Iran; 141 Department of Pharmacology, All India Institute of Medical Sciences, Jodhpur, India; 142 Research Department, Better Vision Foundation Nepal, Kathmandu, Nepal; 143 Ophthalmology and Vision Science, Shahid Beheshti University of Medical Sciences, Tehran, Iran; 144 Social Determinants of Health Research Center, Saveh University of Medical Sciences, Saveh, Iran; 145 Department of Anthropology, Panjab University, Chandigarh, India; 146 Ophthalmology Department, Ministry of Health & Population, Aswan, Egypt; 147 Digestive Diseases Research Institute, Tehran University of Medical Sciences, Tehran, Iran; 148 Department GF Ingrassia, University of Catania, Catania, Italy; 149 University Centre Varazdin, University North, Varazdin, Croatia; 150 National Data Management Center for Health, Ethiopian Public Health Institute, Addis Ababa, Ethiopia; 151 Optometry & Vision Sciences, Zahedan University of Medical Sciences, Zahedan, Iran; 152 Eye Research Center, Mashhad University of Medical Sciences, Mashhad, Iran; 153 Non-communicable Diseases Research Center, Tehran University of Medical Sciences, Tehran, Iran; 154 Department of Medical Laboratory Sciences, Adigrat University, Adigrat, Ethiopia; 155 School of Medicine, Western Sydney University, Campbelltown, NSW Australia; 156 Department of Optometry and Vision Science, University of KwaZulu-Natal, KwaZulu-Natal, South Africa; 157 Global Health Governance Programme, University of Edinburgh, Edinburgh, UK; 158 School of Dentistry, University of Leeds, Leeds, UK; 159 Department of Genetics, Yale University, New Haven, CT USA; 160 Department of Statistics and Econometrics, Bucharest University of Economic Studies, Bucharest, Romania; 161 Medical School, Pham Ngoc Thach University of Medicine, Ho Chi Minh City, Viet Nam; 162 Ophthalmology department, Isfahan University of Medical Sciences, Isfahan, Iran; 163 Department of Neonatology, Case Western Reserve University, Cleveland, OH USA; 164 Department of Population Science and Human Resource Development, University of Rajshahi, Rajshahi, Bangladesh; 165 Multidisciplinary Laboratory Foundation University School of Health Sciences (FUSH), Foundation University, Islamabad, Pakistan; 166 International Center of Medical Sciences Research (ICMSR), Islamabad, Pakistan; 167 Applied Biomedical Research Center, Mashhad University of Medical Sciences, Mashhad, Iran; 168 Biotechnology Research Center, Mashhad University of Medical Sciences, Mashhad, Iran; 169 Department of Medicine, Tehran University of Medical Sciences, Tehran, Iran; 170 Department of Ophthalmology, Harvard Medical School, Boston, MA USA; 171 Ophthalmic Research Center (ORC), Shahid Beheshti University of Medical Sciences, Tehran, Iran; 172 Department of Veterinary Public Health and Preventive Medicine, Usmanu Danfodiyo University, Sokoto, Sokoto, Nigeria; 173 Aier Eye Hospital, Jinan university, Guangzhou, China; 174 1st Department of Ophthalmology, Aristotle University of Thessaloniki, Thessaloniki, Greece; 175 Department of Medicine, University of Crete, Heraklion, Greece; 176 Medical Genomics Research Department, King Abdullah International Medical Research Center, Riyadh, Saudi Arabia; 177 Department of Life Sciences, University of Management and Technology, Lahore, Pakistan; 178 School of Public Health, Zhejiang University, Zhejiang China; 179 Department of Public Health Science, Fred Hutchinson Cancer Research Center, Seattle, WA USA; 180 Macquarie Medical School, Macquarie University, Sydney, NSW Australia; 181 Department of Bioengineering and Therapeutic Sciences, University of California San Francisco, San Francisco, CA USA; 182 Addictology Department, Russian Medical Academy of Continuous Professional Education, Moscow, Russia; 183 School of Medicine, Wuhan University, Wuhan, China

**Keywords:** Epidemiology, Retinal diseases

## Abstract

**Objectives:**

To estimate global and regional trends from 2000 to 2020 of the number of persons visually impaired by diabetic retinopathy and their proportion of the total number of vision-impaired individuals.

**Methods:**

Data from population-based studies on eye diseases between 1980 to 2018 were compiled. Meta-regression models were performed to estimate the prevalence of blindness (presenting visual acuity <3/60) and moderate or severe vision impairment (MSVI; <6/18 to ≥3/60) attributed to DR. The estimates, with 95% uncertainty intervals [UIs], were stratified by age, sex, year, and region.

**Results:**

In 2020, 1.07 million (95% UI: 0.76, 1.51) people were blind due to DR, with nearly 3.28 million (95% UI: 2.41, 4.34) experiencing MSVI. The GBD super-regions with the highest percentage of all DR-related blindness and MSVI were Latin America and the Caribbean (6.95% [95% UI: 5.08, 9.51]) and North Africa and the Middle East (2.12% [95% UI: 1.55, 2.79]), respectively. Between 2000 and 2020, changes in DR-related blindness and MSVI were greater among females than males, predominantly in the super-regions of South Asia (blindness) and Southeast Asia, East Asia, and Oceania (MSVI).

**Conclusions:**

Given the rapid global rise in diabetes and increased life expectancy, DR is anticipated to persist as a significant public health challenge. The findings emphasise the need for gender-specific interventions and region-specific DR healthcare policies to mitigate disparities and prevent avoidable blindness. This study contributes to the expanding body of literature on the burden of DR, highlighting the need for increased global attention and investment in this research area.

## Introduction

Diabetes mellitus (DM) and its complications are a major burden of disease around the world. DM has increased significantly in recent decades and will continue to rise in the next few decades, with a greater burden expected in low-middle income countries (LMICs) [1]. One of the most common microvascular complications of DM is diabetic retinopathy (DR). According to previous large-population based studies and meta-analyses, DR has been recognized as one of the most common causes of blindness and vision impairment among the working-age population; however, this is not true for some countries, such as the United Kingdom, due to the implementation of national DR strategies aimed at identifying and treating patients with this condition [2–9]. The Global Burden of Disease Study (GBD) began 30 years ago to systematically assess and scientifically report on critical health outcomes including DM and its complications. The findings are reported longitudinally and across various populations [10]. In 2020, DR was listed as one of the leading causes of global blindness among those aged 50 years and above [3]. Leasher et al. assessed changes in the prevalence of DR-related blindness and moderate or severe vision impairment (MSVI) from 1990 to 2010 [8]. Findings showed that DR accounted for 2.6% of all blindness and 1.9% of all MSVI in 2010, an increase from 2.1% and 1.3%, respectively, from 1990 [8]. Early detection and treatment interventions for DR can reduce the risk of severe visual loss by approximately 90% [11].

The Lancet Global Health Commission emphasised how improving eye health contributes to achieving the sustainable development goals (SDGs) of improving general health and well-being, reducing poverty and increasing work productivity, and improving education and equity [7]. Due to the unmet need of an ageing and growing population globally, eye health is a major public health concern that requires urgent attention to develop innovative treatments and deliver services on a large scale. Political commitment is necessary to act on eye health, particularly in low-resource settings [7, 12].

The current meta-analysis provides an update of all available population-based studies from 2000 to 2020 to present estimates on the number of people (aged 50 years+) affected by DR-related blindness and DR-related MSVI. Additionally, we investigate the global and regional differences in the prevalence of DR-related blindness and MSVI, and differences by sex.

## Materials/subjects and methods

Preparation of data included first a systematic review of published (between Jan 1, 1980, and Oct 1, 2018) population-based studies of vision impairment and blindness by the Vision Loss and Expert Group (VLEG) that also included gray literature sources. Eligible studies from this review were then combined with data from Rapid Assessment of Avoidable Blindness (RAAB) studies. Data from the US National Health and Nutrition Examination survey and the World Health Organization (WHO) Study on Global Ageing and Adult Health were contributed by the GBD team. More detailed methods are published elsewhere [3, 13] and briefly discussed as follows.

In total, VLEG identified 137 studies and extracted data from 70 studies in their 2010 review, and additional 67 studies in their 2014–18 review. Studies were primarily national and subnational cross-sectional surveys. Additionally, the VLEG commissioned the preparation of 5-year age-disaggregated RAAB data from the RAAB repository. Studies were included if they met the following criteria: visual acuity data had to be measured using a test chart that could be mapped to the Snellen scale, and the sample had to be representative of the population. Self-report of vision loss was excluded. We used International Classification of Diseases 11^th^ (ICD-11) edition criteria for vision impairment, as used by WHO, which categorises people according to vision in the better eye on presentation, in which moderate vision impairment is defined as a visual acuity of 6/60 or better but less than 6/18, severe vision impairment as a visual acuity of 3/60 or better but less than 6/60, and blindness as a visual acuity of less than 3/60 or less than 10° visual field around central fixation (although the visual field definition is rarely used in population-based eye surveys) [14].

First, we separated raw data into vision-loss envelopes for all-cause mild, moderate, and severe vision impairment, and blindness. Data were input into a mixed-effects meta-regression tool developed by the Institute for Health Metrics and Evaluation (IHME) called MR-BRT (meta regression; Bayesian; regularized; trimmed) [15]. Presenting vision impairment was the reference definition for each level of severity. Undercorrected refractive error data were extracted directly from data sources where available, and otherwise calculated by subtracting best-corrected vision impairment from presenting vision impairment prevalence for each level of severity in studies that reported both measures for a given location, sex, age group, and year. All other causes were quantified as part of the best-corrected estimates of vision impairment at each level of severity.

We modeled distance vision impairment and blindness due to the following causes: cataract, undercorrected refractive error, age-related macular degeneration, myopic macular degeneration, glaucoma, diabetic retinopathy, and other causes of vision impairment (in aggregate). Minimum age for inclusion of data for these causes was set at 20 years for cataract and diabetic retinopathy, and 45 years for glaucoma and age-related macular degeneration. Other vision impairment estimates were combined with less prevalent causes of vision impairment to create a residual category (e.g., retinopathy of prematurity, corneal opacities or optic atrophy, trachoma).

We produced location, year, age, and sex-specific estimates of MSVI and blindness using Disease Modeling Meta-Regression (Dismod-MR) 2.1 [16]. The data processing steps are described elsewhere [3]. Briefly, Dismod-MR 2.1 models were run for all vision impairment by severity (moderate, severe, blindness) regardless of cause and, separately, for MSVI and blindness due to each modeled cause of vision impairment (e.g., MSVI due to cataract and blindness due to cataract). Then, models of MSVI due to specific causes were split into moderate and severe estimates using the ratio of overall prevalence in the all-cause moderate presenting vision impairment and severe presenting vision impairment models. Next, prevalence estimates for all causes by severity were scaled to the models of all-cause prevalence by severity. This produced final estimates by age, sex, year, and location for each individual cause of vision impairment by severity. We age-standardized our estimates using the GBD standard population [17].

## Results

According to our estimates from 2020, approximately 1.07 million (95% uncertainty intervals (UIs): 0.76, 1.51) people were blind and nearly 3.28 million (95% UI: 2.41, 4.34) had MSVI globally due to DR (Table 1). An estimated 462,000 males and 611,000 females of all ages, and 368,000 males and 494,000 females aged ≥50 years had DR-related blindness in 2020 (Table 2). The number of males and females (all ages) with DR-related MSVI in 2020 was 1.4 million and 1.8 million, respectively, whereas an estimated 1.3 million and 1.7 million people were aged 50 years and over (Table 3). Higher prevalence rates of DR-related blindness were seen among females aged 60 years and above, with the highest rates observed in people aged >95 years. Higher prevalence rates of DR-related blindness and MSVI were seen among females aged 60 years and above, with the highest rates observed in females aged >95 years.

**Table 1:** Number of people (mean [95% UI]) with DR-related blindness (presenting visual acuity <3/60) or DR-related MSVI (presenting visual acuity <6/18, ≥3/60), the age-standardized prevalence (%) in people of all ages and aged ≥50 years (mean [95% UI]), and the percentage of all blindness or MSVI attributed to DR (95% UI) in 2020 by 7 GBD super-regions.

	DR-related blindness in 2020				DR-related MSVI in 2020		
World Region	2020, Total population (‘000 s)	Number of people (‘000 s) with DR-related blindness in 2020 *(all ages)*	Age-standardized prevalence of DR-related blindness in 2020 (*aged* ≥ *50 years*)	Percentage of all DR-related blindness per region in 2020 (≥50 years)	Number of people (‘000 s) with DR-related MSVI in 2020 (*all ages*)	Age-standardized prevalence of DR-related MSVI in people ≥50 years aged in 2020	Percentage of DR-related MSVI per region in 2020 (≥50 years)
Global	7,890,000	1,074 (763, 1514)	0.05 (0.03, 0.07)	2.50 (1.77, 3.52)	3278 (2409, 4344)	0.16 (0.12, 0.21)	1.11 (0.82, 1.47)
Central Europe, Eastern Europe, and Central Asia	417,291	13 (9, 19)	0.01 (0.01, 0.01)	0.97 (0.67, 1.39)	144 (102, 193)	0.09 (0.07, 0.13)	0.80 (0.57, 1.08)
High-income	1,087,856	161 (116, 227)	0.03 (0.02, 0.04)	5.37 (3.86, 7.55)	420 (308, 558)	0.08 (0.06, 0.11)	1.35 (0.99, 1.80)
Latin America and Caribbean	601,551	254 (185, 347)	0.15 (0.10, 0.21)	6.95 (5.08, 9.51)	451 (333, 598)	0.30 (0.22, 0.40)	1.84 (1.36, 2.45)
North Africa and Middle East	631,727	73 (50, 105)	0.06 (0.04, 0.09)	2.37 (1.63, 3.42)	462 (339, 609)	0.41 (0.30, 0.55)	2.12 (1.55, 2.79)
South Asia	1,841,435	196 (135, 285)	0.05 (0.03, 0.07)	1.65 (1.14, 2.39)	444 (323, 598)	0.13 (0.09, 0.17)	0.46 (0.34, 0.62)
Southeast Asia, East Asia, and Oceania	2,192,710	325 (222, 478)	0.04 (0.03, 0.06)	2.16 (1.48, 3.17)	1190 (862, 1593)	0.18 (0.13, 0.24)	1.43 (1.04, 1.92)
Sub-Saharan Africa	1,114,806	49 (35, 71)	0.03 (0.02, 0.05)	0.98 (0.69, 1.40)	164 (120, 217)	0.14 (0.10, 0.19)	0.81 (0.59, 1.06)

**Table 2:** Number of males and females with DR-related blindness (presenting visual acuity <3/60), and the age-standardized prevalence (% [95% UI]) of DR-related blindness (all ages and people aged ≥50 years) in 2020 by 7 GBD super-regions.

	Total population	Total number of DR-related blindness and age-standardized DR-related blindness in 2020 (all ages)				Total number of DR-related blindness and age-standardized DR-related blindness in people aged 50+ years in 2020			
World Region	2020 total population (000 s)	Number of males with DR-related blindness in 2020	Number of females with DR-related blindness in 2020	Age-standardized prevalence of DR-related blindness in males in 2020	Age-standardized prevalence of DR-related blindness in females in 2020	Number of males (50+ years) with DR-related blindness in 2020	Number of females (50+ years) with DR-related blindness in 2020	Age-standardized prevalence of DR-related blindness in males in 2020	Age-standardized prevalence of DR-related blindness in females in 2020
Global	7,890,000	462.927.18 (325,654.61–652,044.38)	611,103.46 (435,144.17–859,526.29)	0.01 (0.01, 0.02)	0.01 (0.01, 0.02)	367,532.63 (248,081.18–527,880.98)	493,647.89 (340,091.39–705,878.95)	0.04 (0.02, 0.06)	0.05 (0.03, 0.07)
Central Europe, Eastern Europe, and Central Asia	417,291	1755.87 (1093.16–2682.48)	11,965.13 (8,228.75–17,190.34)	0.00 (0.00, 0.00)	0.00 (0.00, 0.00)	1,481.80 (911.96–2301.54)	10,388.59 (7,004.18–15,046.41)	0.00 (0.00, 0.00)	0.01 (0.01, 0.02)
High-income	1,087,856	44,382.55 (30,587.44–64,097.86)	117,013.99 (84,406.34–162,072.26)	0.01 (0.00, 0.01)	0.01 (0.01, 0.02)	38,713.55 (25,822.71–57,277.14)	100,431.86 (71,172.17–138,889.24)	0.02 (0.01, 0.03)	0.04 (0.03, 0.06)
Latin America and Caribbean	601,551	126,678.01 (91,595.33–174,863.61)	127,505.49 (94,172.48–173,729.00)	0.04 (0.03, 0.06)	0.04 (0.03, 0.05)	99,032.99 (67,913.52–139,574.07)	103,802.70 (73,225.22–143,172.95)	0.16 (0.11, 0.22)	0.14 (0.10, 0.20)
North Africa and Middle East	631,727	40,410.14 (27,627.57–58,733.35)	32,864.35 (22,466.72–48,315.39)	0.02 (0.01, 0.02)	0.01 (0.01, 0.02)	33,270.71 (21,679.83–49,856.62)	27,733.97 (18,402.82–42,027.66)	0.07 (0.05, 0.10)	0.06 (0.04, 0.09)
South Asia	1,841,435	81988.27 (56304.46–117390.29)	114658.79 (79601.64–167678.53)	0.01 (0.01, 0.02)	0.02 (0.01, 0.02)	64620.90 (42826.69–94579.40)	87110.02 (57949.08–129720.46)	0.04 (0.03, 0.06)	0.05 (0.04, 0.08)
Southeast Asia, East Asia, and Oceania	2,192,710	148,475.37 (101,487.27–215,934.63)	176,643.96 (120,673.90–260,725.76)	0.01 (0.01, 0.02)	0.01 (0.01, 0.02)	116,365.81 (76,418.03–172,826.78)	144,301.43 (94,401.38–214,160.92)	0.04 (0.02, 0.06)	0.04 (0.03, 0.06)
Sub-Saharan Africa	1,114,806	19236.98 (13185.23–27388.12)	30451.75 (21586.92–43727.97)	0.01 (0.01, 0.01)	0.01 (0.01, 0.01)	14046.86 (9235.32–21084.33)	19879.33 (13645.78–28518.23)	0.03 (0.02, 0.05)	0.04 (0.03, 0.05)

**Table 3:** Number of males and females with DR-related MSVI, and the age-standardized prevalence (% [95% uncertainty intervals (UIs)]) of DR-related MSVI (all ages and people aged ≥ 50 years) in 2020 by 7 GBD super-regions.

	Total population	Total number of DR-related MSVI and age-standardized DR-related MSVI in 2020 (all ages)				Total number of DR-related MSVI and age-standardized DR-related MSVI in people aged 50+ years in 2020			
World Region	2020, total population (100 s)	Number of males with DR-related MSVI in 2020	Number of females with DR-related MSVI in 2020	Age-standardized prevalence of DR-related MSVI in males in 2020	Age-standardized prevalence of DR-related MSVI in females in 2020	Number of males (50+ years) with DR-related MSVI in 2020	Number of females (50+ years) with DR-related MSVI in 2020	Age-standardized prevalence of DR-related MSVI in males in 2020	Age-standardized prevalence of DR-related MSVI in females in 2020
Global	7,890,000	1,434,563.64 (1,058,082.45–1,906,706.91)	1,843,470.26 (1,354,361.39–2,434,417.31)	0.04 (0.03, 0.05)	0.04 (0.03, 0.05)	1,283,152.12 (929,301.13–1,730,971.73)	1,662,787.39 (1,207,905.41–2,222,537.30)	0.15 (0.11, 0.20)	0.17 (0.12, 0.22)
Central Europe, Eastern Europe, and Central Asia	417,291	50,221.20 (35,532.76–67,893.80)	94,204.50 (66,934.75–124,937.93)	0.02 (0.01, 0.03)	0.02 (0.02, 0.03)	46,096.08 (32,051.61–63,189.24)	88,358.52 (62,275.75–118,849.35)	0.08 (0.06, 0.11)	0.10 (0.07, 0.14)
High-income	1,087,856	168,636.22 (124,963.48–224,391.14)	251,401.81 (183,394.45–336,375.21)	0.02 (0.01, 0.02)	0.02 (0.02, 0.03)	151,688.80 (108,728.71–204,628.93)	233,889.33 (167,461.31–317,886.32)	0.07 (0.05, 0.10)	0.09 (0.06, 0.12)
Latin America and Caribbean	601,551	210,400.26 (155,349.78–282,311.45)	240,993.75 (177,872.69–318,631.42)	0.07 (0.06, 0.10)	0.07 (0.05, 0.10)	184,619.39 (133,297.98–249,704.02)	211,681.86 (153,838.77–284,165.76)	0.30 (0.22, 0.41)	0.29 (0.21, 0.39)
North Africa and Middle East	631,727	209,853.63 (154,123.75–277,168.06)	252,518.04 (186,029.20–333,427.73)	0.09 (0.07, 0.12)	0.11 (0.08, 0.15)	180,252.63 (130,165.51–244,813.42)	219,082.90 (158,244.71–295,314.20)	0.37 (0.27, 0.51)	0.45 (0.33, 0.61)
South Asia	1,841,435	195,237.80 (141,348.63–261,562.12)	249,304.72 (181,409.73–336,125.23)	0.03 (0.02, 0.04)	0.03 (0.02, 0.05)	170,861.07 (121,996.86–231,790.95)	217,902.35 (155,497.63–296,733.22)	0.11 (0.08, 0.15)	0.14 (0.10, 0.19)
Southeast Asia, East Asia, and Oceania	2,192,710	529,736.41 (382,298.34–712,821.22)	660,634.29 (480,541.67–876,817.85)	0.04 (0.03, 0.05)	0.04 (0.03, 0.06)	490,582.61 (352,372.31–665,085.13)	613,025.86 (442,398.51–825,577.13)	0.17 (0.12, 0.23)	0.19 (0.14, 0.25)
Sub-Saharan Africa	1,114,806	70,478.12 (51,543.64–93,966.89)	94,413.15 (69,139.60–124,855.35)	0.03 (0.02, 0.04)	0.04 (0.03, 0.05)	59,051.54 (42,153.34–80,066.25)	78,846.57 (56,570.77–107,274.46)	0.13 (0.10, 0.18)	0.15 (0.11, 0.20)

DR caused 2.50% (95% UI: 1.77, 3.52) of blindness in 2020 worldwide. Regionally, the highest percentage of all DR-related blindness was found in Latin America and Caribbean (6.95% [95% UI: 5.08, 9.51]) and High-Income super-regions (5.37% [95% UI: 3.86, 7.55]) (Table 1). The super-regions with the lowest percentage of all DR-related blindness were Central Europe, Eastern Europe, and Central Asia (0.97% [95% UI: 0.67, 1.39]), and Sub-Saharan Africa (0.98% [95% UI: 0.69, 1.40]). DR caused 1.11% (95% UI: 0.82, 1.47) of MSVI in 2020 worldwide. North Africa and Middle East (2.12% [95% UI: 1.55, 2.79]), and Latin America and Caribbean (1.84% [95% UI: 1.36, 2.45]) were super-regions with the highest percentage of all MSVI due to DR (Table 1).

**Fig. 1:**
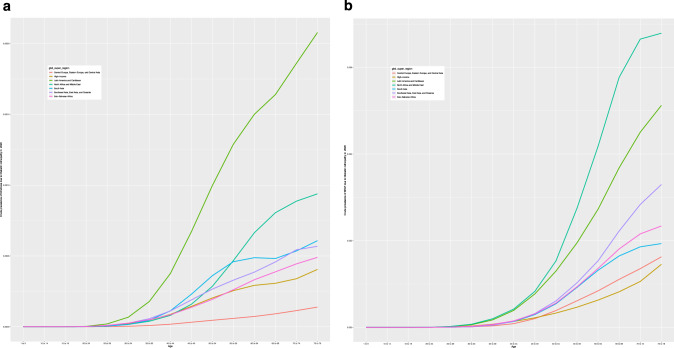
Prevalence of blindness and moderate to severe visual impairment (MSVI) due to diabetic retinopathy (DR) in 2020 across seven global burden of disease (GBD) super-regions. Crude prevalence of blindness and MSVI due to DR in 2020 by age, across seven world GBD super-regions. **a** Crude prevalence of blindness due to DR in 2020 by seven world GBD super-regions by age. The graph demonstrates an increase in prevalence with age, with notable variations between super-regions. The super-regions are represented by different coloured lines. **b** Crude prevalence of MSVI in 2020 by seven world GBD super-regions by age. Similar to (**a**), the prevalence increases with age, highlighting disparities among different super-regions. Each super-region is depicted by a distinct coloured line.

In 2020, the global age-standardized prevalence of DR-related blindness in those aged ≥50 years was 0.05% (95% UI: 0.03, 0.07) and 0.16% (95% UI: 0.12, 0.21) for DR-related MSVI (Table 1). The super-region with the highest age-standardized prevalence of DR-related blindness was Latin American and Caribbean (0.15% [95% UI: 0.10, 0.21]). The lowest age-standardized prevalence of DR-related blindness in 2020 was in Central Europe, Eastern Europe, and Central Asia (0.01% [95% UI: 0.01, 0.01]). The super-regions with the highest age-standardized prevalence of DR-related MSVI in 2020 were North Africa and Middle East (0.41% [95% UI: 0.30, 0.55]), and Latin America and the Caribbean (0.30% [95% UI: 0.22, 0.40]). The lowest estimates were found in the High-Income (0.08% [95% UI: 0.06, 0.11]) and Central Europe, Eastern Europe, and Central Asia (0.09% [95% UI: 0.07, 0.13]) super-regions (Table 1). Figure 1 presents the crude prevalence of blindness and MSVI due to DR in 2020 across super-regions.

Between 2000 and 2020, the global percentage change in age-standardized prevalence of DR-related blindness among adults (≥50 years) showed different trends for males and females (Supplementary file, Table S1). For males, there was a minimal decrease of −0.10% (95% UI: −0.54, 0.34), while females experienced an increase of +12.89% (95% UI: 12.40, 13.38). An overall increase in the age-standardized prevalence of DR-related blindness among adults aged ≥50 years (both sexes) was found in South Asia (+25.66% [95% UI: 25.07, 26.24]), Southeast Asia, East Asia and Oceania (+15.36% [95% UI: 14.80, 15.92]) and Sub-Saharan Africa (+2.47% [95% UI: 2.01, 2.94]). An increase of +14.92% (95% UI: 14.39, 15.45) in age-standardized prevalence of DR-related blindness in South Asia from 2000 to 2020 was observed for males, whiles females experienced even greater gains with a rise of +34.68% (95% UI: 34.04, 35.32). In Southeast Asia, East Asia, and Oceania, the increase in age-standardized prevalence of DR-related blindness from 2000 to 2020 was +3.43% (95% UI: 2.94, 3.91) for males, compared to +26.34% (95% UI: 25.72, 26.97) for females. In Sub-Saharan Africa, although the overall age-standardized prevalence of DR-related blindness from 2000 to 2020 increased, a decrease was found among males (−12.46% [95% UI: −12.87, −12.04]) compared to females (+16.79% [95% UI: 16.27, 17.30]). All other super-regions demonstrated a decrease in the age-standardized prevalence of DR-related blindness (≥50 years) from 2000 to 2020 overall. In Central Europe, Eastern Europe and Central Asia, the age-standardized prevalence of DR-related blindness decreased by −21.99% (95% UI: −22.41, −21.58) for males compared to −3.15% (95% UI: −3.61, −2.70) for females. In Latin America and Caribbean, a decrease of −20.74% (95% UI: −21.06, −20.41) was observed in males, with a smaller decrease (−5.49% [95% UI: −5.86, −5.11]) among females. In the High-Income super-region, a reduction of −15.73% (95% UI: −16.13,−15.32) and −8.46% (95% UI: −8.83, −8.09) was found in males and females, respectively. Supplementary file contains Figs. (S1–S4) illustrating the total number of cases (males and females) with DR-related blindness and MSVI between 2000 and 2020, for all 21 GBD world regions, including the global total for comparison.

From 2000 to 2020, there was a decrease in the global percentage change in age-standardized prevalence of DR-related MSVI (≥50 years) among males (−0.93% [95% UI: −1.29, −0.56]), while females experienced an increase (+3.62% [95% UI: 3.25, 3.99]). Between 2000 and 2020, the super-region of Southeast Asia, East Asia, and Oceania showed an increase in the age-standardized prevalence of DR-related MSVI for both males (+1.17%, [95% UI: 0.79, 1.55]) and females (+3.33%, [95% UI: 2.95, 3.71]). In Sub-Saharan Africa, there was a decrease in the age-standardized prevalence of DR-related MSVI among males (−1.98%, [95% UI:−2.34, −1.63]), whereas females experienced an increase (+1.06%, [95% UI: 0.69, 1.42]). All other super-regions demonstrated a decrease in the age-standardized prevalence of DR-related MSVI (≥50 years) between 2000 and 2020 for both sexes. The super-region of North Africa and the Middle East showed the most notable decline in age-standardized DR-related MSVI for both sexes (−15.35% [−15.66, −15.05]). Among males, there was a decrease of −16.43% (95% UI: −16.73, −16.12), while females exhibited a −14.57% (95% UI: −14.88, −14.26) decrease (Supplementary file, Table S2).

The global percentage change in crude prevalence for DR-related blindness between 2000 and 2020 was +1.41% (95% UI: −0.96, 1.85) in males compared to a + 13.32% (95% UI: 12.83, 13.80) increase in females, and +7.90% (95% UI: 7.43, 8.36) overall. The percentage change in crude prevalence of DR-related MSVI was also higher among females (+3.56% (95% UI: 3.18, 3.93)) compared to males (+1.31% (95% UI: 0.93, 1.69)) globally (Supplementary file, Tables S1, 2).

## Discussion

Although DR remains highly prevalent, the figures from 2020 show a slight decrease compared to those reported in 2010 [8]. In 2020, DR accounted for 2.5% of global blindness and 1.1% of MSVI, down from 2.6% and 1.9%, respectively, in 2010. Leasher et al. also showed that the highest age-standardized prevalence of DR-related blindness and MSVI was in the super-regions of North Africa/Middle East, Sub-Saharan Africa, and South Asia, while the lowest prevalence was in High-Income regions [8]. An increase in the numbers of people with DR-related blindness and MSVI with a relatively unchanged age-standardized prevalence from 2010 to 2020 may be attributed to the increasing population and average age in most regions, coupled with falling death rates.

Our study found that DR-related blindness has increased more among females than males in almost all super-regions. The largest sex-related inequalities were found in South Asia, Southeast Asia, East Asia and Oceania, and Sub-Saharan Africa. Though there are age-adjusted declines in DR prevalence for some super-regions, the overall global crude prevalence of both DR-related blindness and DR-related MSVI for males, females, and overall has increased globally due to aging and growth of the population. These figures represent the true burden of disease with which governments must contend.

The factors contributing to these gender disparities are multifaceted. One possible contributing factor is the difference in average life expectancy between women and men. As women tend to have a longer lifespan, they are consequently at greater risk of developing DM and DR. In LMICs, women may have poorer access to healthcare services compared to men [18, 19]. Other factors that may contribute to disparities in eye health include, lack of access to information and resources, and lower literacy among females compared to males [20–22]. Pregnancy is another factor that can accelerate the progression of DR in women [23]. Finally, DR has been linked to intake of the retinal carotenoids lutein and zeaxanthin, and women are thought to have lower retinal levels of lutein and zeaxanthin [24, 25]. The difference in retinal levels of lutein and zeaxanthin between men and women may be due to several factors including hormones, dietary patterns, and variances in metabolic processes [25]. Factors such as smoking might vary between women and men, contributing to differences in retinal levels. This requires further investigation to ascertain the precise causes behind the observed differences in retinal levels between men and women. Action is needed to improve female care and reduce the burden of DR-related blindness and MSVI.

Teo et al. estimated that there would be 103.12 million people with DR, 28.54 million people with vision-threatening DR, and 18.83 million people with clinically significant macular oedema in 2020 [26]. They found that the North America and Caribbean (NAC) and Middle East and North Africa (MENA) showed significantly higher prevalence of DR compared to other regions [26]. Similarly, our results show that the Latin America and Caribbean and North Africa, and Middle East super-regions demonstrated the highest prevalence of DR-related blindness and MSVI. This may be attributed to several factors such as limited access to quality healthcare services, increased DM cases, and inadequate management of DM. Although DR is estimated to affect over 100 million people globally, our data from 2020 suggests that less than 1.1 million are currently blind and less than 3.3 million are visually impaired. Compared to the 2010 data, 834,000 people were blind whereas 3.7 million were visually impaired [8]. The decline in the number of people with MSVI from 2010, despite an increase in DR-related blindness may be due to advancements in medical technology and treatments for DR. They play a role in preventing the progression of the disease to more severe stages, hence reducing the number of individuals with MSVI. Additionally, increased awareness about DM and its ocular complications might lead to earlier detection and intervention, which could prevent or mitigate MSVI cases despite the rise in DR-related blindness.

Blindness and MSVI can have a profound impact on quality of life, impairing both mental and physical health, and social independence [27]. As reported in the GBD Study 2019, blindness and low vision was ranked eighth (contributing 3·8% [95% UI 3·0, 4·9]) of all years lived with disability (YLDs) in people aged 50–69 years [13]. Among people aged 70 years and older, blindness and low vision was ranked fourth (contributing 6·4% [5·4, 7·4] of all YLDs) [13]. Furthermore, blindness and MSVI are associated with reduced economic, educational, and employment opportunities [28–30]. Economic productivity at the individual, family, community, and national level is important to sustainable development. An inability to work can diminish the productive capacity of the economy by reducing the workforce. Illness and disability can contribute to productivity losses through absenteeism from work, reduced productivity while at work or unemployment, including job loss and early retirement [28–31]. The Lancet Global Health Commission on Global Eye Health assessed the overall relative reduction in employment by working-aged people with blindness and MSVI [31]. They found that the global average relative reduction in employment of people with vision impairment was estimated to be 30.2% [31]. Since blindness and MSVI can have a large economic impact globally, more data on the employment status of people living with blindness and MSVI in all world regions, especially, LMICs needs to be available. Future research should explore more specifically how DR-related blindness and MSVI affect productivity losses and if there are relevant differences by sex.

We reviewed the literature to determine the economic burden of DR globally. According to UK estimates, DR has an annual cost of £379 million($476 million) for cases linked to type 2 DM, and almost £14 million ($17.6 million) for cases related to type 1 DM [32]. Economic modeling in the UK suggests that reducing the prevalence of type 2 DM-related DR by just 1% each year could save the UK economy £150 million ($188.6 million) by 2050 [32]. The estimated economic burden of DR in the United States is $0.5 billion [33], $3.91 billion in Germany [34], and $3.5 to 6.4 billion in the Latin America and the Caribbean region [35]. Further exploration of the economic burden in all world regions is necessary for agenda setting and policy planning in the future.

### Strengths

The VLEG populates and curates the Global Vision Database, a continuously updated, comprehensive, online database storing worldwide ophthalmic epidemiological information, including DR. By considering data from Jan 1st 1980 to Oct 1st 2018, the study covers a significant period, allowing for the assessment of trends and changes over time. The inclusion of gray literature enriches the database with unpublished data yet valuable data.

Our report provides an update on the worldwide and regional estimates for DR-related blindness and MSVI, including the changing patterns over time. It demonstrates that considerable regional differences and sex inequalities exist, highlighting areas that require particular attention such as low resource settings. These findings could aid further region-specific DR healthcare policies to prevent vision impairment, especially among females in the future.

### Limitations

This meta-analysis has some limitations, such as potential publication bias and heterogeneity across studies. Due to the paucity of data across low burden regions, we may be over/under-estimating DR overall prevalence. While visual acuity is an important measure of visual function, it is not the only measure, and it is important to consider other methods of measuring visual impairment such as contrast sensitivity when assessing the prevalence of vision impairment. Nonetheless, our findings highlight the ongoing burden of DR-related vision impairment and underscore the need for effective prevention and management strategies.

Early detection and timely treatment are essential for preventing avoidable DR-related blindness and MSVI [36, 37]. Between 2000 and 2020, high-income countries have made good progress in terms of reducing their DR-related blindness/MSVI which may be linked to improved risk factor control and advances in their screening and treatment services [7, 38, 39]. Despite this success, screening and treatment services still remain a challenge for super-regions such as Latin America (high prevalence of all DR-related blindness and MSVI ≥50 years old) [40]. While Sub-Saharan Africa might be anticipated to have a higher burden of DR compared to regions such as Latin America and Caribbean, Middle East, and North Africa, differences in population demographics, genetics, lifestyle, and DM management approaches contribute to varied prevalence rates. Under-reporting and insufficient data availability further complicate assessing the true extent of the issue. While healthcare resources are limited in Sub-Saharan Africa, certain areas within the region may have stronger healthcare infrastructure or targeted interventions that improve DR management compared to other LMICs. The global burden of DR is expected to remain high through 2045, disproportionately affecting countries in the Middle East and North Africa, and the Western Pacific [26]. Delivering innovative DR prevention and treatment strategies to improve global eye health is necessary. Screening for DR would also be much improved by the existence of population DM registers. Finally, our findings suggest the need for region-specific healthcare policies aimed at preventing vision loss, particularly among females.

Supplemental material is available at Eye’s website.

## Summary

### What was known before

- Globally, in 2020, 1.07 million people were blind, and nearly 3.28 million were visually impaired by diabetic retinopathy.

### What this study adds

- The contribution of diabetic retinopathy and moderate and severe vision impairment (MSVI) by region and the change in this contribution between 2000 and 2020. The change in global age-standardized prevalence of DR-related blindness and MSVI between 2000 and 2020 and the differences by sex and region.

### Supplementary information

Table S1

Table S2

Fig S1: Number of males (all ages) with MSVI due to Diabetic retinopathy in 2000 and 2020 by 21 GBD world regions

Fig S2: Number of females (all ages) with MSVI due to Diabetic retinopathy in 2000 and 2020 by 21 GBD world regions

Fig S3: Number of males (all ages) with blindness due to Diabetic retinopathy in 2000 and 2020 by 21 GBD world regions

Fig S4: Number of females (all ages) with blindness due to Diabetic retinopathy in 2000 and 2020 by 21 GBD world regions

Appendix: Contributions by Authors

## Data Availability

The data that support the findings of this study are not openly available due to reasons of sensitivity and are available from the coordinator of the Vision Loss Expert Group (Professor Rupert Bourne; rb@rupertbourne.co.uk) upon reasonable request. Data are located in controlled access data storage at Anglia Ruskin University.
