# Supplementary material for: Global estimates on the number of people blind or visually impaired by diabetic retinopathy: a meta-analysis from 2000 to 2020
Source: Eye (Lond). 2024 Jun 27;38(11):2047–57. doi: 10.1038/s41433-024-03101-5 (PMC11269692; doi:10.1038/s41433-024-03101-5)
Supplement: Supplementary file 1 — Table S1 [file 41433_2024_3101_MOESM1_ESM.docx]

**Table S1: Percentage change in crude prevalence, number of cases and age-standardized prevalence of DR-related blindness (presenting visual acuity <3/60) in adults aged 50 years and older between 2000 and 2020 by 7 GBD super-regions**

|  | **Crude Prevalence** | | | **Number of cases (‘000s)** | | | **Age-standardized prevalence** | | |
| --- | --- | --- | --- | --- | --- | --- | --- | --- | --- |
| **World Region** | Male (%, 95% UI) | Female (%, 95% UI) | Both (%, 95% UI) | Male (n, 95% UI) | Female (n, 95% UI) | Both (n, 95% UI) | Male (%, 95% UI) | Female (%, 95% UI) | Both (%, 95% UI) |
| **Global** | +1.41 (-0.96, 1.85) | +13.32 (12.83, 13.80) | +7.90 (7.43, 8.36) | +78.85 (78.06, 79.63) | +99.06 (98.20, 99.92) | +89.90 (89.08, 90.72) | -0.10 (-0.54, 0.34) | +12.89 (12.40, 13.38) | +6.97 (6.51, 7.43) |
| **Central Europe, Eastern Europe and Central Asia** | -20.00 (-20.45, -19.55) | -0.32 (-0.78, 0.15) | -4.55 (-5.00, -4.10) | +3.41 (2.84, 3.99) | +23.33 (22.76, 23.90) | +20.44 (19.87, 21.00) | -21.99 (-22.41, -21.58) | -3.15 (-3.61, -2.70) | -7.27 (-7.70, -6.83) |
| **High income countries** | -11.25 (-11.68, -10.82) | -5.51 (-5.89, -5.13) | -8.07 (-8.46, -7.68) | +30.60 (29.96, 31.24) | +31.61 (31.09, 32.14) | +31.33 (30.78, 31.88) | -15.73 (-16.13, -15.32) | -8.46 (-8.83, -8.09) | -11.57 (-11.94, -11.20) |
| **Latin America and Caribbean** | -20.55 (-20.88, -20.22) | -4.23 (-4.61, -3.84) | -13.19 (-13.54, -12.84) | +56.56 (55.91, 57.21) | +96.03 (95.24, 96.82) | +74.55 (73.84, 75.26) | -20.74 (-21.06, -20.41) | -5.49 (-5.86, -5.11) | -13.77 (-14.12, -13.42) |
| **North Africa and Middle East** | -5.21 (-5.67, -4.75) | -2.59 (-3.07, -2.12) | -4.05 (-4.51, -3.58) | +96.94 (95.99, 97.89) | +102.69 (101.71, 103.68) | +99.52 (98.55, 100.48) | -1.44 (-1.90, -0.98) | -0.51 (-0.99, -0.03) | -1.20 (-1.67, -0.74) |
| **South Asia** | +15.50 (14.95, 16.04) | +34.97 (34.30, 35.63) | +26.20 (25.59, 26.81) | +117.97 (116.93, 119.00) | +172.86 (171.52, 174.19) | +146.43 (145.24, 147.61) | +14.92 (14.39, 15.45) | +34.68 (34.04, 35.32) | +25.66 (25.07, 26.24) |
| **Southeast Asia, East Asia and Oceania** | +6.67 (6.16, 7.19) | +27.21 (26.58, 27.83) | +17.12 (16.55, 17.69) | +110.92 (109.91, 111.94) | +159.48 (158.21, 160.76) | +135.30 (134.15, 136.45) | +3.43 (2.94, 3.91) | +26.34 (25.72, 26.97) | +15.36 (14.80, 15.92) |
| **Sub-Saharan Africa** | -16.09 (-16.49, -15.68) | +12.78 (12.28, 13.27) | -1.42 (-1.87, -0.97) | +48.45 (47.73, 49.17) | +118.78 (117.81, 119.74) | +82.90 (82.07, 83.74) | -12.46 (-12.87, -12.04) | +16.79 (16.27, 17.30) | +2.47 (2.01, 2.94) |
