# Supplementary material for: Global estimates on the number of people blind or visually impaired by diabetic retinopathy: a meta-analysis from 2000 to 2020
Source: Eye (Lond). 2024 Jun 27;38(11):2047–57. doi: 10.1038/s41433-024-03101-5 (PMC11269692; doi:10.1038/s41433-024-03101-5)
Supplement: Supplementary file 2 — Table S2 [file 41433_2024_3101_MOESM2_ESM.docx]

**Table S2: Percentage change in crude prevalence, number of cases and age-standardized prevalence of DR-related MSVI (presenting visual acuity <6/18, ≥3/60) in adults aged 50 years and older between 2000 and 2020 by 7 GBD super-regions**

|  | **Crude Prevalence** | | | **Number of cases (‘000s)** | | | **Age-standardized prevalence** | | |
| --- | --- | --- | --- | --- | --- | --- | --- | --- | --- |
| **World Region** | Male (%, 95% UI) | Female (%, 95% UI) | Both (%, 95% UI) | Male (n, 95% UI) | Female (n, 95% UI) | Both (n, 95% UI) | Male (%, 95% UI) | Female (%, 95% UI) | Both (%, 95% UI) |
| **Global** | +1.31 (0.93, 1.69) | +3.56 (3.18, 3.93) | +2.55 (2.18, 2.93) | +78.68 (78.01, 79.34) | +81.92 (81.26, 82.58) | +80.49 (79.83, 81.15) | -0.93 (-1.29, -0.56) | +3.62 (3.25, 3.99) | +1.58 (1.21, 1.94) |
| **Central Europe, Eastern Europe and Central Asia** | -2.09 (-2.48, -1.70) | -2.20 (-2.58, -1.83) | -2.52 (-2.90, -2.14) | +27.00 (26.49, 27.51) | +20.99 (20.53, 21.46) | +22.99 (22.51, 23.47) | -5.26 (-5.63, -4.89) | -3.01 (-3.38, -2.64) | -4.16 (-4.53, -3.78) |
| **High income countries** | +2.07 (1.69, 2.45) | -0.97 (-1.35, -0.60) | -0.25 (-0.62, 0.13) | +50.20 (49.64, 50.76) | +37.93 (37.41, 38.46) | +42.51 (41.98, 43.05) | -6.41 (-6.75, -6.06) | -4.09 (-4.44, -3.73) | -5.67 (-6.02, -5.32) |
| **Latin America and Caribbean** | -0.43 (-0.80, -0.06) | +0.23 (-0.13, 0.60) | -0.09 (-0.45, 0.28) | +96.22 (95.49, 96.95) | +105.16 (104.41, 105.91) | +100.90 (100.16, 101.63) | -1.99 (-2.34, -1.63) | -1.80 (-2.15, -1.44) | -1.93 (-2.29, -1.58) |
| **North Africa and Middle East** | -20.19 (-20.49, -19.90) | -16.48 (-16.79, -16.17) | -18.19 (-18.49, -17.89) | +65.82 (65.21, 66.43) | +73.80 (73.16, 74.44) | +70.11 (69.47, 70.74) | -16.43 (-16.73, -16.12) | -14.57 (-14.88, -14.26) | -15.35 (-15.66, -15.05) |
| **South Asia** | -12.20 (-12.53, -11.87) | -2.18 (-2.56, -1.80) | -6.66 (-7.02, -6.31) | +65.69 (65.07, 66.32) | +97.76 (97.00, 98.52) | +82.26 (81.57, 82.95) | -14.48 (-14.79, -14.16) | -4.71 (-5.06, -4.35) | -9.12 (-9.45, -8.79) |
| **Southeast Asia, East Asia and Oceania** | +3.42 (3.02, 3.82) | +3.54 (3.15, 3.93) | +3.62 (3.23, 4.01) | +104.49 (103.70, 105.29) | +111.21 (110.42, 112.00) | +108.17 (107.38, 108.96) | +1.17 (0.79, 1.55) | +3.33 (2.95, 3.71) | +2.34 (1.96, 2.72) |
| **Sub-Saharan Africa** | -4.72 (-5.08, -4.36) | -2.68 (-3.05, -2.31) | -3.22 (-3.59, -2.86) | +68.55 (67.91, 69.20) | +88.79 (88.08, 89.51) | +79.56 (78.88, 80.24) | -1.98 (-2.34, -1.63) | +1.06 (0.69, 1.42) | +0.01 (-0.36, 0.37) |
