# Supplementary figures and images for: Global estimates on the number of people blind or visually impaired by diabetic retinopathy: a meta-analysis from 2000 to 2020
Source: Eye (Lond). 2024 Jun 27;38(11):2047–57. doi: 10.1038/s41433-024-03101-5 (PMC11269692; doi:10.1038/s41433-024-03101-5)

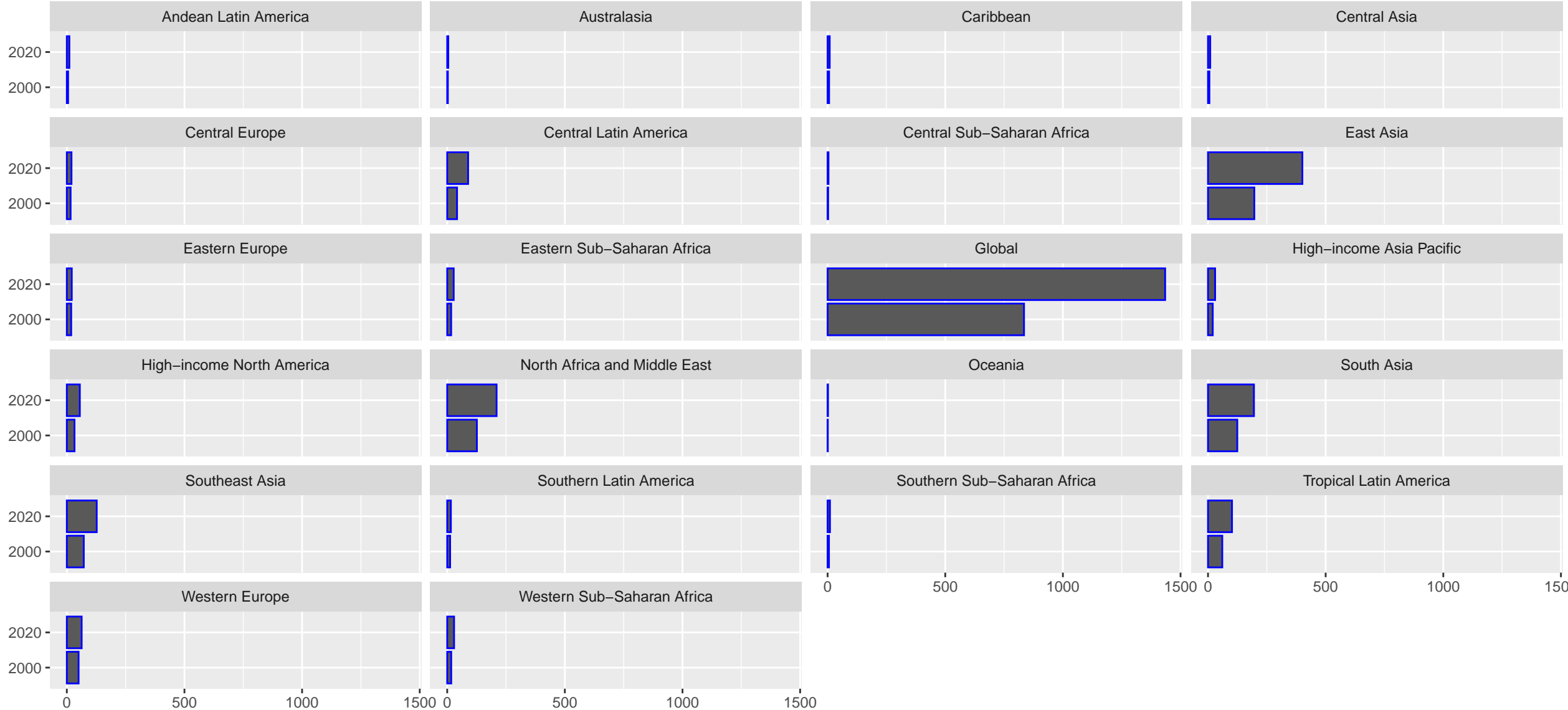

Number of Males with MSVI due to Diabetic retinopathy in 2000 and 2020 by world region of all ages

Supplement: Supplementary file 3 — Fig S1: Number of males (all ages) with MSVI due to Diabetic retinopathy in 2000 and 2020 by 21 GBD world regions [file 41433_2024_3101_MOESM3_ESM.pdf]

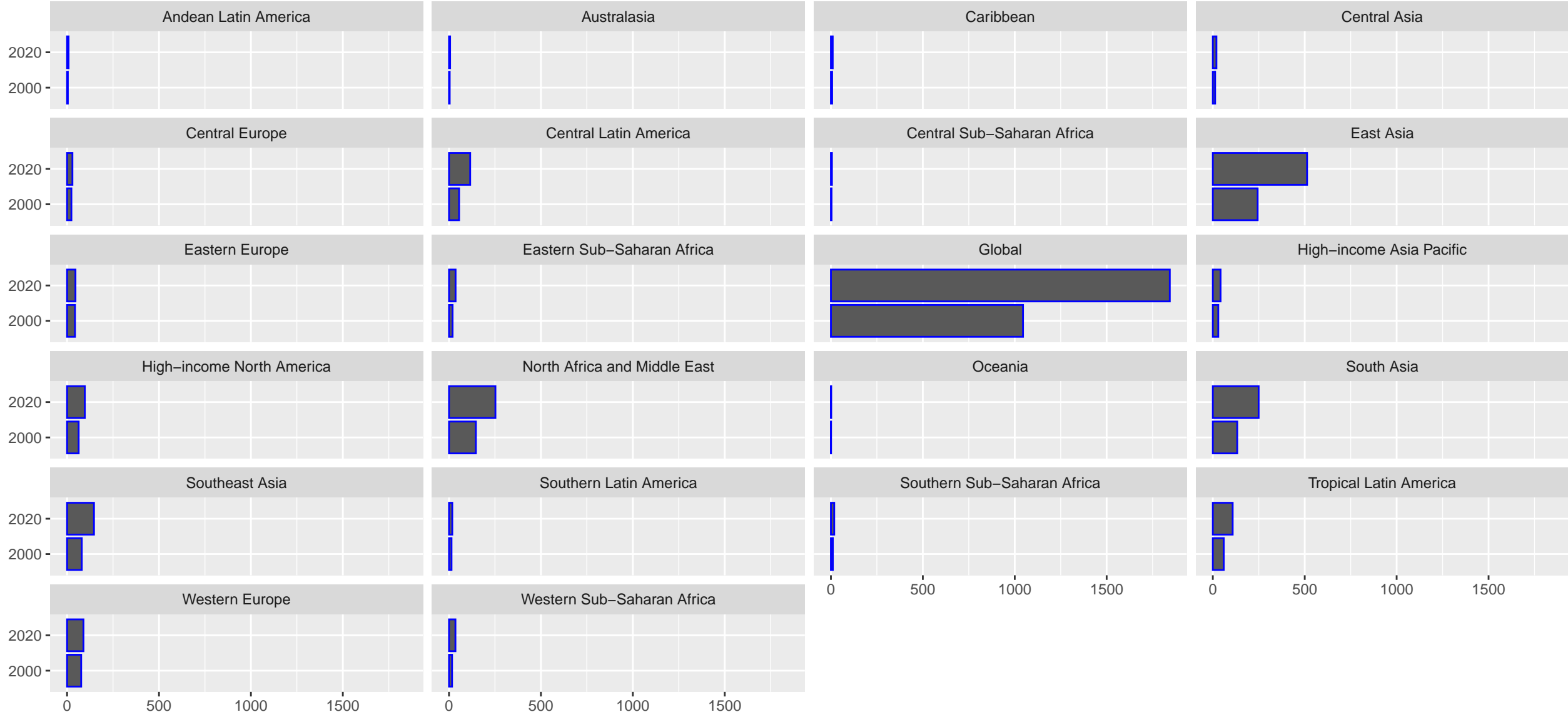

Number of females with MSVI due to Diabetic retinopathy in 2000 and 2020 by world region of all ages

Supplement: Supplementary file 4 — Fig S2: Number of females (all ages) with MSVI due to Diabetic retinopathy in 2000 and 2020 by 21 GBD world regions [file 41433_2024_3101_MOESM4_ESM.pdf]

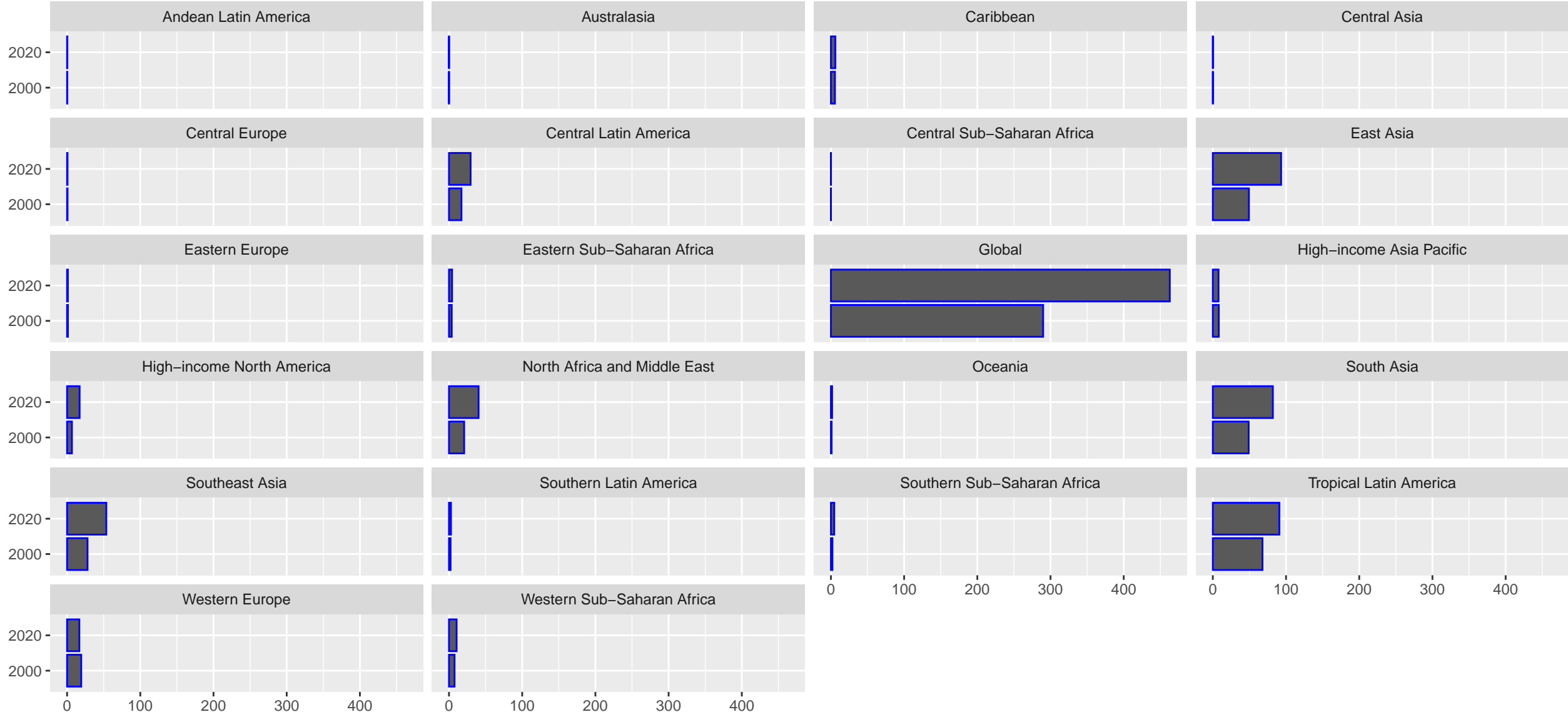

Number of Males with blindness due to Diabetic retinopathy in 2000 and 2020 by world region of all ages

Supplement: Supplementary file 5 — Fig S3: Number of males (all ages) with blindness due to Diabetic retinopathy in 2000 and 2020 by 21 GBD world regions [file 41433_2024_3101_MOESM5_ESM.pdf]

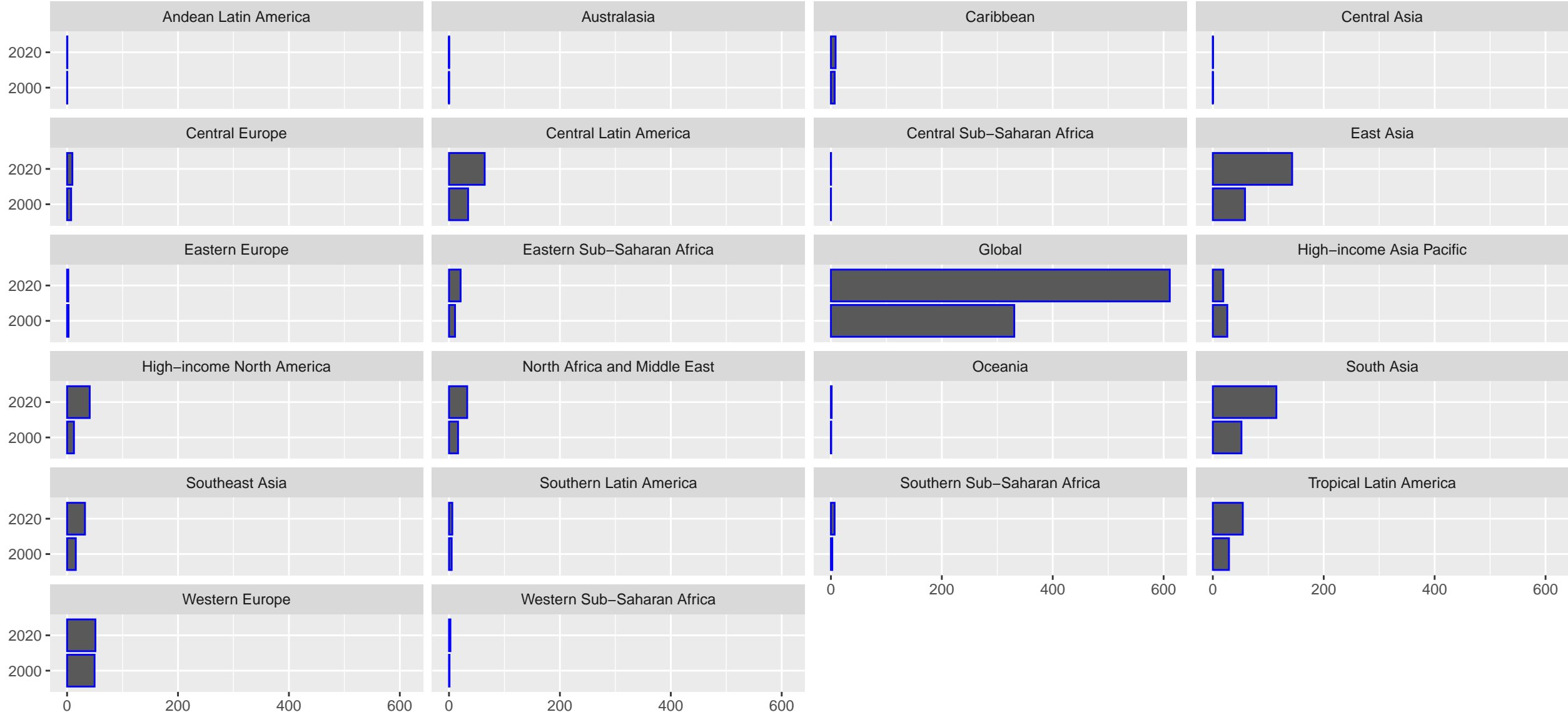

Number of Females with blindness due to Diabetic retinopathy in 2000 and 2020 by world region of all ages

Supplement: Supplementary file 6 — Fig S4: Number of females (all ages) with blindness due to Diabetic retinopathy in 2000 and 2020 by 21 GBD world regions [file 41433_2024_3101_MOESM6_ESM.pdf]
