# Supplementary material for: Global estimates on the number of people blind or visually impaired by diabetic retinopathy: a meta-analysis from 2000 to 2020
Source: Eye (Lond). 2024 Jun 27;38(11):2047–57. doi: 10.1038/s41433-024-03101-5 (PMC11269692; doi:10.1038/s41433-024-03101-5)
Supplement: Supplementary file 7 — Appendix: Contributions by Authors [file 41433_2024_3101_MOESM7_ESM.docx]

**Appendix: Contributions by Authors**

# GBD 2019 Blindness and Vision Impairment Collaborators

### Providing data or critical feedback on data sources

Bright Opoku Ahinkorah, Ayman Ahmed, Louay Almidani, Hubert Amu, Sofia Androudi, Jalal Arabloo, Ahmed Y Azzam, Freddie Bailey, Mehmet Firat Baran, Mainak Bardhan, Till Winfried Bärnighausen, Amadou Barrow, Mukharram Bikbov, Rupert Bourne, Tasanee Braithwaite, Paul Svitil Briant, Katie Curran, Xiaochen Dai, Thanh Chi Do, Thao Huynh Phuong Do, Klara Georgieva Dokova, Michael Ekholuenetale, Mohammad Hassan Emamian, Hossein Farrokhpour, Ali Fatehizadeh, Lorenzo Ferro Desideri, João M Furtado, Mesay Dechasa Gudeta, Sapna Gupta, Veer Bala Gupta, Vivek Kumar Gupta, Mehdi Hosseinzadeh, John J Huang, Sheikh Mohammed Shariful Islam, Jost B Jonas, Charity Ehimwenma Joshua, Vidya Kadashetti, Himal Kandel, Sudarshan Khanal, Kewal Krishan, Janet L Leasher, Stephen S Lim, Mohammed Magdy Abd El Razek, Andrea Maugeri, Awoke Misganaw, Ali H Mokdad, Christopher J L Murray, Uchechukwu Levi Osuagwu, Shahina Pardhan, Jay Patel, Shrikant Pawar, Tunde Peto, Hoang Tran Pham, Umar Saeed, Tabassom Sedighi, Maryam Shayan, Aminu Shittu, Jaimie D Steinmetz, Hugh R Taylor, Fotis Topouzis, Aristidis Tsatsakis, Muhammad Umair, Theo Vos, Mikhail Sergeevich Zastrozhin, and Zhi-Jiang Zhang.

### Developing methods or computational machinery

Hubert Amu, Aleksandr Y Aravkin, Ahmed Y Azzam, Mehmet Firat Baran, Rupert Bourne, Paul Svitil Briant, Kaleb Coberly, Xiaochen Dai, Thanh Chi Do, Mehdi Emamverdi, Ali Fatehizadeh, Lorenzo Ferro Desideri, Hamidreza Hasani, Mehdi Hosseinzadeh, Jost B Jonas, Mohammed Magdy Abd El Razek, Ali H Mokdad, Hamed Momeni-Moghaddam, Christopher J L Murray, Hoang Tran Pham, Umar Saeed, Jaimie D Steinmetz, Muhammad Umair, Theo Vos, and Peng Zheng.

### Providing critical feedback on methods or results

Bright Opoku Ahinkorah, Hamid Ahmadieh, Ayman Ahmed, Ahmad Samir Alfaar, Louay Almidani, Hubert Amu, Sofia Androudi, Jalal Arabloo, Mulu Tiruneh Asemu, Ahmed Y Azzam, Nayereh Baghcheghi, Freddie Bailey, Mehmet Firat Baran, Mainak Bardhan, Till Winfried Bärnighausen, Amadou Barrow, Pankaj Bhardwaj, Mukharram Bikbov, Rupert Bourne, Tasanee Braithwaite, Paul Svitil Briant, Katrin Burkart, Luis Alberto Cámera, Katie Curran, Omid Dadras, Xiaochen Dai, Amin Dehghan, Berecha Hundessa Demessa, Mengistie Diress, Thanh Chi Do, Thao Huynh Phuong Do, Klara Georgieva Dokova, Michael Ekholuenetale, Muhammed Elhadi, Mohammad Hassan Emamian, Mehdi Emamverdi, Hossein Farrokhpour, Ali Fatehizadeh, Lorenzo Ferro Desideri, João M Furtado, Mesfin Gebrehiwot, Fariba Ghassemi, Mesay Dechasa Gudeta, Sapna Gupta, Veer Bala Gupta, Vivek Kumar Gupta, Mehdi Harorani, Hamidreza Hasani, Golnaz Heidari, Mehdi Hosseinzadeh, John J Huang, Sheikh Mohammed Shariful Islam, Nilofer Javadi, Aida Jimenez-Corona, Mohammad Jokar, Jost B Jonas, Charity Ehimwenma Joshua, Vidya Kadashetti, Himal Kandel, Rimple Jeet Kaur, Sudarshan Khanal, Zahra Khorrami, Judy E Kim, Hamid Reza Koohestani, Kewal Krishan, Janet L Leasher, Stephen S Lim, Mohammed Magdy Abd El Razek, Vahid Mansouri, Andrea Maugeri, Tomislav Mestrovic, Awoke Misganaw, Ali H Mokdad, Hamed Momeni-Moghaddam, Sara Momtazmanesh, Christopher J L Murray, Hadush Negash, Uchechukwu Levi Osuagwu, Shahina Pardhan, Jay Patel, Shrikant Pawar, Ionela-Roxana Petcu, Tunde Peto, Hoang Tran Pham, Mohsen Pourazizi, Ibrahim Qattea, Mosiur Rahman, Umar Saeed, Mohammad Amin Salehi, Maryam Shayan, Aminu Shittu, Yao Tan, Fotis Topouzis, Muhammad Umair, Theo Vos, Hong Xiao, Yuyi You, and Mikhail Sergeevich Zastrozhin.

### Drafting the work or revising it critically for important intellectual content

Bright Opoku Ahinkorah, Hamid Ahmadieh, Ayman Ahmed, Ahmad Samir Alfaar, Hubert Amu, Jalal Arabloo, Mulu Tiruneh Asemu, Ahmed Y Azzam, Freddie Bailey, Mehmet Firat Baran, Mainak Bardhan, Till Winfried Bärnighausen, Amadou Barrow, Mukharram Bikbov, Rupert Bourne, Tasanee Braithwaite, Katie Curran, Amin Dehghan, Berecha Hundessa Demessa, Thanh Chi Do, Bruce B Duncan, Muhammed Elhadi, Mohammad Hassan Emamian, Mehdi Emamverdi, Ali Fatehizadeh, Arthur G Fernandes, David S Friedman, João M Furtado, Mesfin Gebrehiwot, Fariba Ghassemi, Mesay Dechasa Gudeta, Sapna Gupta, Veer Bala Gupta, Vivek Kumar Gupta, Billy Randall Hammond, Golnaz Heidari, Sheikh Mohammed Shariful Islam, Nilofer Javadi, Aida Jimenez-Corona, Jost B Jonas, Vidya Kadashetti, Himal Kandel, Hengameh Kasraei, Rimple Jeet Kaur, Sudarshan Khanal, Zahra Khorrami, Judy E Kim, Kewal Krishan, Janet L Leasher, Mohammed Magdy Abd El Razek, Vahid Mansouri, Andrea Maugeri, Tomislav Mestrovic, Awoke Misganaw, Ali H Mokdad, Hamed Momeni-Moghaddam, Sara Momtazmanesh, Christopher J L Murray, Hadush Negash, Uchechukwu Levi Osuagwu, Shahina Pardhan, Jay Patel, Shrikant Pawar, Ionela-Roxana Petcu, Tunde Peto, Hoang Tran Pham, Mohsen Pourazizi, Umar Saeed, Amirhossein Sahebkar, Mohammad Amin Salehi, Aminu Shittu, Yao Tan, Hugh R Taylor, Muhammad Umair, Theo Vos, Mikhail Sergeevich Zastrozhin, and Zhi-Jiang Zhang.

### Managing the estimation or publications process

Ahmed Y Azzam, Rupert Bourne, Katie Curran, Thanh Chi Do, Ali Fatehizadeh, Mohammed Magdy Abd El Razek, Ali H Mokdad, Christopher J L Murray, Tunde Peto, Hoang Tran Pham, Ibrahim Qattea, Muhammad Umair, Theo Vos, and Mikhail Sergeevich Zastrozhin.

Vision Loss Expert Group of the Global Burden of Disease Study

### Providing data or critical feedback on data sources

Alessandro Arrigo, Mukharram M Bikbov, Rupert R A Bourne, Tasanee Braithwaite, Alain Bron, Ching-Yu Cheng, Maria Vittoria Cicinelli, Katie Curran, Monte A Del Monte, Joshua R Ehrlich, Arthur Fernandes, Seth Flaxman, David Friedman, João M Furtado, Gus Gazzard, M Elizabeth Hartnett, Jost B Jonas, Rim Kahloun, John H Kempen, Moncef Khairallah, Rohit C Khanna, Judy E Kim, Van Charles Lansingh, Janet Leasher, Nicolas Leveziel, Kovin S Naidoo, Vinay Nangia, Michal Nowak, Konrad Pesudovs, Tunde Peto, Pradeep Ramulu, Serge Resnikoff, Tabassom Sedighi, Ian Tapply, Hugh Taylor, Fotis Topouzis, Miltiadis Tsilimbaris, Ya Xing Wang, Ningli Wang

### Developing methods or computational machinery

Rupert R A Bourne, Jost B Jonas, Ian Tapply

### Providing critical feedback on methods or results

Alessandro Arrigo, Mukharram M Bikbov, Rupert R A Bourne, Tasanee Braithwaite, Katie Curran, Monte A Del Monte, Arthur Fernandes, David Friedman, João M Furtado, M Elizabeth Hartnett, Jost B Jonas, Rim Kahloun, John H Kempen, Judy E Kim, Janet Leasher, Konrad Pesudovs, Tunde Peto, Serge Resnikoff, Ian Tapply, Ningli Wang

### Drafting the work or revising it critically for important intellectual content

Alessandro Arrigo, Mukharram M Bikbov, Rupert R A Bourne, Tasanee Braithwaite, Katie Curran, Monte A Del Monte, Arthur Fernandes, M Elizabeth Hartnett, Jost B Jonas, Judy E Kim, Janet Leasher, Konrad Pesudovs, Tunde Peto

### Managing the estimation or publications process

Rupert R A Bourne, Katie Curran, Jost B Jonas
